# Development of an mHealth App by Experts for Queer Individuals’ Sexual-Reproductive Health Care Services and Needs: Nominal Group Technique Study

**DOI:** 10.2196/59963

**Published:** 2024-08-21

**Authors:** Raikane James Seretlo, Hanlie Smuts, Mathildah Mpata Mokgatle

**Affiliations:** 1 Department of Public Health, School of Health Care Sciences Sefako Makgatho Health Sciences University Tshwane South Africa; 2 Department of Informatics, Faculty of Engineering, Built Environment and Information Technology University of Pretoria Tshwane South Africa

**Keywords:** experts, mobile health app, mHealth app, digital health care, telemedicine, digital innovation, sexual-reproductive health care services and needs, lesbian, gay, bisexual, transgender, queer, intersex, asexual individuals, and related identities, LBQTQIA+, queer individuals, nominal group technique, mobile phone

## Abstract

**Background:**

Queer individuals continue to be marginalized in South Africa; they experience various health care challenges (eg, stigma, discrimination, prejudice, harassment, and humiliation), mental health issues (eg, suicide and depression), and an increased spread of HIV or AIDS and sexually transmitted illnesses (STIs; chlamydia, gonorrhea, and syphilis). Mobile health (mHealth) apps have the potential to resolve the health care deficits experienced by health care providers when managing queer individuals and by queer individuals when accessing sexual-reproductive health care services and needs, thus ensuring inclusivity and the promotion of health and well-being. Studies have proven that the nominal group technique (NGT) could be used to solve different social and health problems and develop innovative solutions. This technique ensures that different voices are represented during decision-making processes and leads to robust results.

**Objective:**

This study aims to identify important contents to include in the development of an mHealth app for addressing the sexual-reproductive health care services and needs of queer individuals.

**Methods:**

We invited a group of 13 experts from different fields, such as researchers, queer activists, sexual and reproductive health experts, private practicing health care providers, innovators, and private health care stakeholders, to take part in a face-to-face NGT. The NGT was conducted in the form of a workshop with 1 moderator, 2 research assistants, and 1 principal investigator. The workshop lasted approximately 2 hours 46 minutes and 55 seconds. We followed and applied 5 NGT steps in the workshop for experts to reach consensus. The main question that experts were expected to answer was as follows: *Which content should be included in the mHealth app for addressing sexual-reproductive health care services and needs for queer individuals?* This question was guided by user demographics and background, health education and information, privacy and security, accessibility and inclusivity, functionality and menu options, personalization and user engagement, service integration and partnerships, feedback and improvement, cultural sensitivity and ethical considerations, legal and regulatory compliance, and connectivity and data use.

**Results:**

Overall, experts voted and ranked the following main icons: menu options (66 points), privacy and security (39 points), user engagement (27 points), information hub (26 points), user demographics (20 points), connectivity (16 points), service integration and partnerships (10 points), functionalities (10 points), and accessibility and inclusivity (7 points).

**Conclusions:**

Conducting an NGT with experts from different fields, possessing vast skill sets, knowledge, and expertise, enabled us to obtain targeted data on the development of an mHealth app to address sexual-reproductive health care services and needs for queer individuals. This approach emphasized the usefulness of a multidisciplinary perspective to inform the development of our mHealth app and demonstrated the future need for continuity in using this approach for other digital health care innovations and interventions.

## Introduction

### Background

The literature shows that most countries are focusing on curbing the spread of sexually transmitted illnesses (STIs) [1-3] by promoting sexual and reproductive health care (SRH) among a vast number of patients. Various public health agencies and organizations, such as the World Health Organization (WHO) and the Joint United Nations Programme on HIV/AIDS, and others are supporting the prioritization of queer individuals’ health and well-being. For example, the WHO formulates guidelines, offers technical assistance, and undertakes research to assist countries in developing and strengthening inclusive health policies and structures that promote the health and well-being of all people, irrespective of sexual orientation, gender identity, expression, and sex characteristics [4]. In addition, Frescura et al [5] stated that United Nations Programme on HIV/AIDS has set additional ambitious targets for 2025, including 95% of all people who are HIV positive knowing their status, 95% of all individuals with HIV receiving prolonged antiretroviral medication, and 95% of every recipient of antiretroviral therapy having viral suppression. Furthermore, enhancing the health and well-being of all people is a central focus of the 2030 Agenda for Sustainable Development. This is evident in targets aimed at eradicating diseases such as AIDS [6]. As a result, countries are expected to develop policies to curb STIs.

In Africa, SRH is also primarily prioritized as part of universal health coverage; however, policies and legal hurdles, limiting gender norms, and gender-based inequities all pose challenges to delivering and accessing quality SRH services in low- or middle-income nations [7]. This situation exists regardless of objective 2 of the Africa Health Strategy 2016-2030, which calls for a reduction in morbidity and an end to preventable mortality from communicable diseases such as AIDS, ensuring equitable access to comprehensive and integrated SRH [8]. The South African National Lesbian, Gay, Bisexual, Transgender, and Intersex (LGBTI) HIV Plan, 2017 to 2022, aimed to provide HIV, STI, and tuberculosis services to LGBTI people through 5 interconnected packages: health, empowerment, human rights, psychosocial support, and assessment [9].

However, in South Africa, SRH services and needs (SRHSN) for queer individuals are either generalized or inadequately addressed. This is evidenced by the continuation of high rates of the spread of STIs among lesbian, gay, bisexual, transgender, questioning, intersex, asexual, and related identities (LGBTQIA+) individuals in the country [10-12]. In addition, several South African studies have shown that queer individuals continue to experience various STIs such as asymptomatic *Neisseria gonorrhoeae*, *Chlamydia trachomatis* [10,12], syphilis [11], and bacterial STI burden. Furthermore, Malefo et al [13] showed that the prevalence of asymptomatic STIs among men who have sex with men was high in South Africa, with discrete bacterial infections detected. According to the study by Khozah and Nunu [14], factors such as clinical settings, punishing laws, and the accessibility of services tailored to sexual and gender minority groups contribute to a generally low average use of SRH.

Several studies found a lack of skills, medical education, and knowledge among health care providers (HCPs) regarding health matters related to queer individuals [15-18]. However, in a South African study focused on motivational interviewing for reducing risky sexual behaviors, most of the participants stated that the sessions had a positive impact on the change and reduction of risky sexual behaviors, specifically by promoting the use of condoms and minimizing the number of multiple sexual partners [19]. Furthermore, skills and knowledge are not the only challenges in the health care sector; HCPs also continue to illtreat, stigmatize, prejudice, discriminate, harass, and judge queer individuals seeking health care services in South Africa, sub-Saharan African regions, and countries globally [20-26].

Unless these challenges are addressed, queer individuals’ health and well-being will continue to be at risk, resulting in the high prevalence and incidence of STIs [27]. South African queer individuals will continue to face extensive challenges, such as societal stigma; homophobic violence, specifically corrective rape; and high rates of STIs, particularly HIV or AIDS [28]. These social challenges hinder queer individuals from accessing and using SRHSN freely.

### The State of Technology Use

In a ubiquitous computerized society, technology is considered a basic and crucial component of our lives [29]. Furthermore, the use of mobile health (mHealth) apps by HCPs globally has increased remarkably [30]. For patients and HCPs alike, mHealth could enhance access to information and also promote communication on healthy living and the management of diseases [31]. Mickan et al [32] highlighted that HCPs benefit from a variety of mobile devices and apps, with the most notable benefits being greater access to point-of-care tools, which have been shown to support better clinical decision-making and outcomes. Therefore, mHealth apps can centralize all of a patient’s medication-specific data, providing a much more logical process for teaching patients how to manage their illnesses and provide self-care [33]. The WHO supports the use of digital health interventions and introduced a guideline that emphasizes that health systems need to harness the heightened visibility and availability of information through the use of mobile phones, tablets, and computers to enhance people’s health and vital services [34]. In addition, the guideline stresses the importance of reaching out to populations considered vulnerable and guaranteeing that digital health care does not expose them to danger in any way [34].

The South African National Department of Health developed a web-based medium connecting the public health workforce to relevant, high-quality continuing professional development opportunities and resources [35]. This web-based platform provides outstanding health care to patients, addresses health care professionals’ need to enhance their abilities and competencies, and executes strategies to shape effective managers and health practitioners [35]. However, this web-based platform does not include details about how HCPs should address queer individuals SRHSN.

These discrete policies and innovative interventions do not stipulate what types of SRHSN HCPs should provide and render to queer individuals; therefore, this research study aimed to identify essential content for inclusion in an mHealth app to address the SRHSN of queer individuals. All the challenges queer individuals experience could be addressed by using innovative technologies such as mHealth apps.

## Methods

### Overview

To determine what content needs to be included in an mHealth app for addressing the SRHSN of queer individuals, we facilitated a face-to-face nominal group technique (NGT) expert workshop at a nongovernmental organization clinic in the Gauteng Province of South Africa. The NGT expert workshop was conducted in February 2024. This NGT expert workshop incorporated 2 design science research steps (suggestion and development) [36]. During the workshop, experts were guided through 4 main NGT stages, as discussed by Mullen et al [37] and designed by Delbecq et al [38].

### Study Population and Recruitment

We sampled 13 experts who voluntarily agreed to participate in our workshop. The workshop criteria of the experts included persons with experience, skills, knowledge, and expertise in SRH, LGBTQI+, and digital innovation. The experts were identified by searching on social media platforms, such as Instagram, LinkedIn, university websites, and ResearchGate, using the search string “SRH, SRH researchers, digital innovation, queer, LGBTQIA+.” Recruitment of the experts occurred over approximately 3 months from December 2023 to February 2024 through email invitations to an initial sample of 22 potential experts; we followed up with calls and emails with those who did not respond. In total, 13 experts agreed to participate; each expert was sent an email with the workshop information and a Google Doc to provide their availability. On the date of the workshop, each participant was given a consent form before data collection commenced (refer to Table 1 for participant demographics). The study used convenience sampling; thus, participants who met the study criteria were easily accessible, resided within the Gauteng province, were available on the date set for the NGT workshop, and were voluntarily willing to participate in the NGT expert workshop [39].

**Table 1:** Experts’ demographic data.

Expert number	Age (y)	Sex	Preferred pronouns	Race^a^	Marital status	Focus area
E1	31	Male	He or him	African	Single	Sexuality, LGBTQIA+^b^, masculinity, and gender
E2	28	Female	She or her	African	Single	Innovative marketing
E3	55	Female	She or her	African	Single	Sexually transmitted illnesses and key populations
E4	39	Male	He or him	African	Single	HIV, tuberculosis, and key populations
E5	35	Male	He or him	African	Married	Health communication and LGBTQIA+
E6	29	Male	He or him	African	Single	Key population research
E7	29	Female	She or her	African	Married	Primary health care
E8	24	Male	He or him	African	Single	Data analytics
E9	34	Female	She or her	African	Single	Psychosocial sciences
E10	27	Female	She or Her	Coloured	Single	Communications
E11	55	Male	He or him	White	Single	Sexual-reproductive health and LGBTQIA+
E12	48	Male	He or him	African	Married	Obstetrics and gynecology and researcher
E13	57	Female	She or her	African	Single	Sexual-reproductive health

^a^Race was self-reported by the participants.

^b^LGBTQIA+: lesbian, gay, bisexual, transgender, queer, intersex, asexual, and related identities.

Table 1 presents the demographics of the experts who participated in the NGT workshop. Among the 13 experts, the majority were male (n=7, 54%) and the minority were female (n=6, 46%); in terms of race, 11 (85%) were African, 1 (8%) was White, and 1 (8%) was of the Coloured racial group. Of the 13 participants, 10 (77%) were single and 3 (23%) were married.

### Instruments for the NGT

The principal investigator conferred with the prospective mobile app software developer before the NGT experts’ workshop and shared the research question to be posed during the workshop, namely, *Which content should be included in the mHealth app to address sexual-reproductive health care services and needs for queer individuals?* The following pillars helped answer the main research questions during the workshop: user demographics and background, health education and information, privacy and security, accessibility and inclusivity, functionality and menu options, personalization and user engagement, service integration and partnerships, feedback and improvement, cultural sensitivity and ethical considerations, legal and regulatory compliance, and connectivity and data use.

Our NGT expert workshop had 1 moderator who conducted the entire workshop following the study by Yahaya et al [40], thereby ensuring bias was avoided by the principal investigator, and assisted with the completion of the consent forms and the smooth running of the workshop. In addition, 2 research assistants participated to ensure that all the experts’ discussions and views were noted on the flipchart boards using markers. Finally, the principal investigator explained the purpose of the workshop, shared information on the pillars, handed out small cards containing written NGT steps whenever experts were moving to each step, and recorded the workshop session with a digital recorder.

### Implementation of the NGT

We used a workshop program to facilitate the NGT expert workshop, thus achieving the purpose of the workshop effectively (program provided in Textbox 1). The workshop lasted approximately 2 hours 46 minutes and 55 seconds.

**Nominal group technique program.**

**Time and activity**

- 09:00 AM: Welcome
- 09:10 AM: Introductions
- 09:30 AM: Purpose and presentation of the findings
- 10:00 AM: Moderator preparation and orientation of experts
- 10:10 AM: Silent generation
- 10:30 AM: Tea break
- 11:00 AM: Round robin
- 11:30 AM: Clarification
- 12:30 PM: Voting and ranking
- 13:10 PM: A vote of thanks and conclusion

In this section, we present the program for the NGT expert workshop, including its duration and the activities conducted during the workshop. The workshop commenced at 09:00 AM with introductions and purpose and presentation of the findings; the first NGT step commenced at 10:10 AM, and the last step was completed at 12:30 PM, followed by a vote of thanks and closure at 13:10 PM.

The workshop moderator facilitated the workshop by first ensuring the experts understood the details in the consent form, guided them through the demographic data section, and answered all the questions raised by the experts up until the signing of the consent form. The attendees signed informed consent forms before the workshop commenced and after all questions from the experts regarding details about the consent form had been clarified. Thereafter, the principal investigator shared small cards describing the first step with all the experts, and the moderator explained what was written on the card, clarifying the activities expected of the experts. The first step was silent generation, whereby the experts were given sticky notes and pens to write down their ideas for answering the workshop or study question and aligning their answers to the guiding pillars. This step lasted 20 minutes, and the experts were not allowed to talk to each other until the time had elapsed. The experts were provided with differently colored sticky notes to group their ideas in silence. The second step, called the round robin, followed, during which the moderator explained what was expected of the experts. The information in the cards clearly indicated that the experts were allowed to share their previously written down ideas with the entire group, while other experts did not interject and critique during a presentation. Each expert had 10 minutes to present their ideas, and other experts were not allowed to add new ideas that might be influenced by the presenting expert. Experts were asked to answer the question, *Which content should be included in the mHealth app to address sexual-reproductive health care services and needs for queer individuals?* The question was supposed to be answered by all the experts, aligning their answers to the shared pillars. After each expert had presented, they handed their sticky notes to the research assistants and principal investigator, and all ideas were captured on a flipchart board using a black marker. A similar process ensued until all 13 experts had completed their presentations. The third NGT step was clarification, which allowed experts to discuss each other’s ideas and identify any similarities, meaning the experts were allowed to question each other and ask for clarification. The discussion session took approximately 1 hour. Finally, the voting and ranking step followed, where all experts were allowed to start looking at all the raw data of the ideas written on the flipchart boards and posted on the wall. The moderator continued to facilitate the session, and 1 research assistant, together with the principal investigator, used a red marker to start marking the grouping of ideas shared and suggested by all the experts. Discussions and arguments about which subthemes to be prioritize continued until the experts reached a consensus (findings presented in Table 2). This step lasted approximately 1.5 hours until a consensus was reached. The moderator continued maintaining order and ensured that all the experts were protected and given a fair chance to argue and vote. The experts were allowed to vote through a show of hands as each subheading was presented, and the moderator counted the votes. The experts voted for whichever contents they believed essential for inclusion in the mHealth app.

**Table 2:** Detailed summary of the final findings including pillars, headings, and subheading with their breakdown ranking and scoring.

Pillar	Headings and subheadings	Ranking and scoring
User demographics	Recording of personal data for profile creation Pronouns such as him, her, they, their, or them Sexual identity such as male, female, binary, or nonbinary Gender orientation such as gay, bisexual, transgender, and so on Age Ability to find locations and health care facilities within different areas Specialized health care facilities Nongovernmental facilities such as POP-INN	11 09
Features	Contact support information Web-based consultation Artificial intelligence assistant or chat bot Suggestion box Call back and email request Videos Clips Graphics Share button Share button for success stories	11 11 10 09 09 08 08
Accessibility and inclusivity	Ensuring inclusivity and easy access by different people Specifically, people with disabilities	07
Connectivity	Ensuring free app access Offline app access through sponsorships Ensuring accommodative of people without smart phones Use of USSID^a^	10 06
Privacy and security	Protection of the user’s data Record keeping Provide users online login details with passwords User’s backup data Agreements between users Avail end user agreement Ensure safeguard of the users Ensure protection of the user’s login details Change of passwords regularly Passwords change reminders for the users	10 10 09 08 02
Functionalities	Push notifications Such as SMS reminders	10
Service integration and partnerships	Health services Health care providers Laboratories Medical aids Private and public health care facilities (which will help with appointments and referrals)	10
User engagement	Ensure referral to other health care providers Option for referral to other health care providers Frequently asked questions Option for frequently asked questions and known facts Support groups Create option for support and peer groups	10 09 08
Information hub	Health education HIV (PrEP^b^ and PEP^c^) STI^d^ Contraceptives Abortions Lubricants and condoms Option of indication when to consult doctors when having complications Gender affirming services Hormonal services and information Gender reassignment services and information Lifestyle education Sex toys use and cleanliness. Dietary advice	11 09 06

^a^USSID: Unstructured Supplementary Service Data.

^b^PrEP: pre-exposure prophylaxis.

^c^PEP: postexposure prophylaxis.

^d^STI: sexually transmitting illness.

Textbox 2 presents a detailed summary of the steps that guided the NGT expert workshop session. The steps included preparations and orientation by the moderator, silent generation, round robin, clarification, voting, and ranking.

**Steps that guided the nominal group technique expert workshop.**

**Steps and details**

1. Moderator preparation and orientation of experts: The moderator explained the consent form to the experts, guided them through the demographic section, answered all questions arising from the consent form, and oriented the experts on how the workshop would run. Shared information with the experts on the pillars that are to be used during the workshop to guide them answering the research question.
2. Silent generation: Experts were given sticky notes and pens and asked to answer the question, “Which SRHSN content should be included in the mHealth app to address SRHSN for queer individuals?” The question was supposed to be answered by all experts, aligning their answers to the shared pillars.
3. Round robin: The experts were given 10 minutes each to share the ideas and suggestions they had written on sticky notes. The experts were allowed to clarify their ideas briefly.
4. Clarification: The experts were allowed to discuss and ask each other questions based on the shared ideas and suggestions. Similar ideas were combined, and duplicates removed as the experts discussed their ideas.
5. Voting and ranking: The moderator asked the experts to examine the recorded ideas on the flipcharts, select their top 10, rank them according to their priority, and indicate which should be first and last in the mobile health app.

### Data Analysis

**Figure 1:**
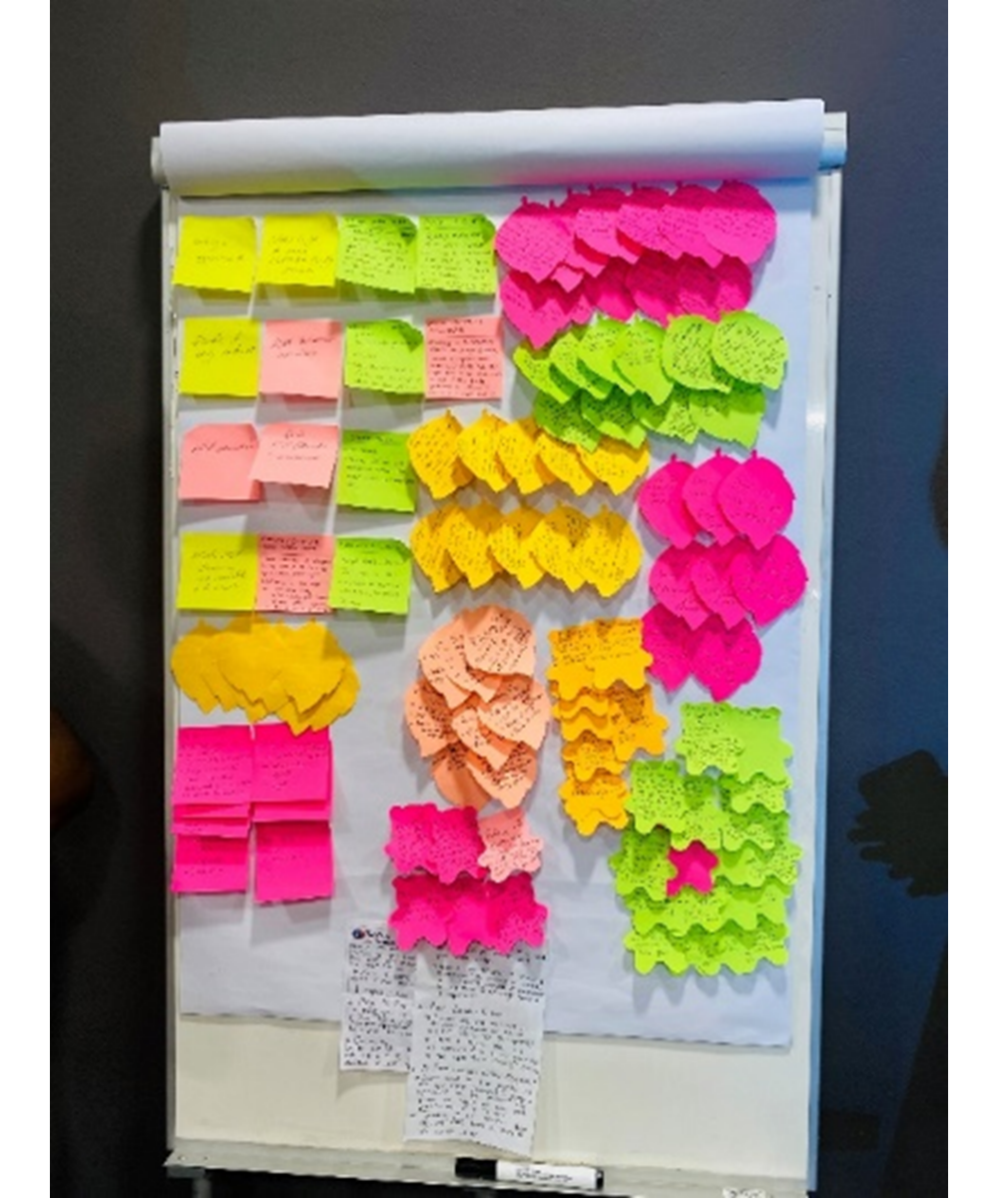
Generated ideas on sticky notes.

**Figure 2:**
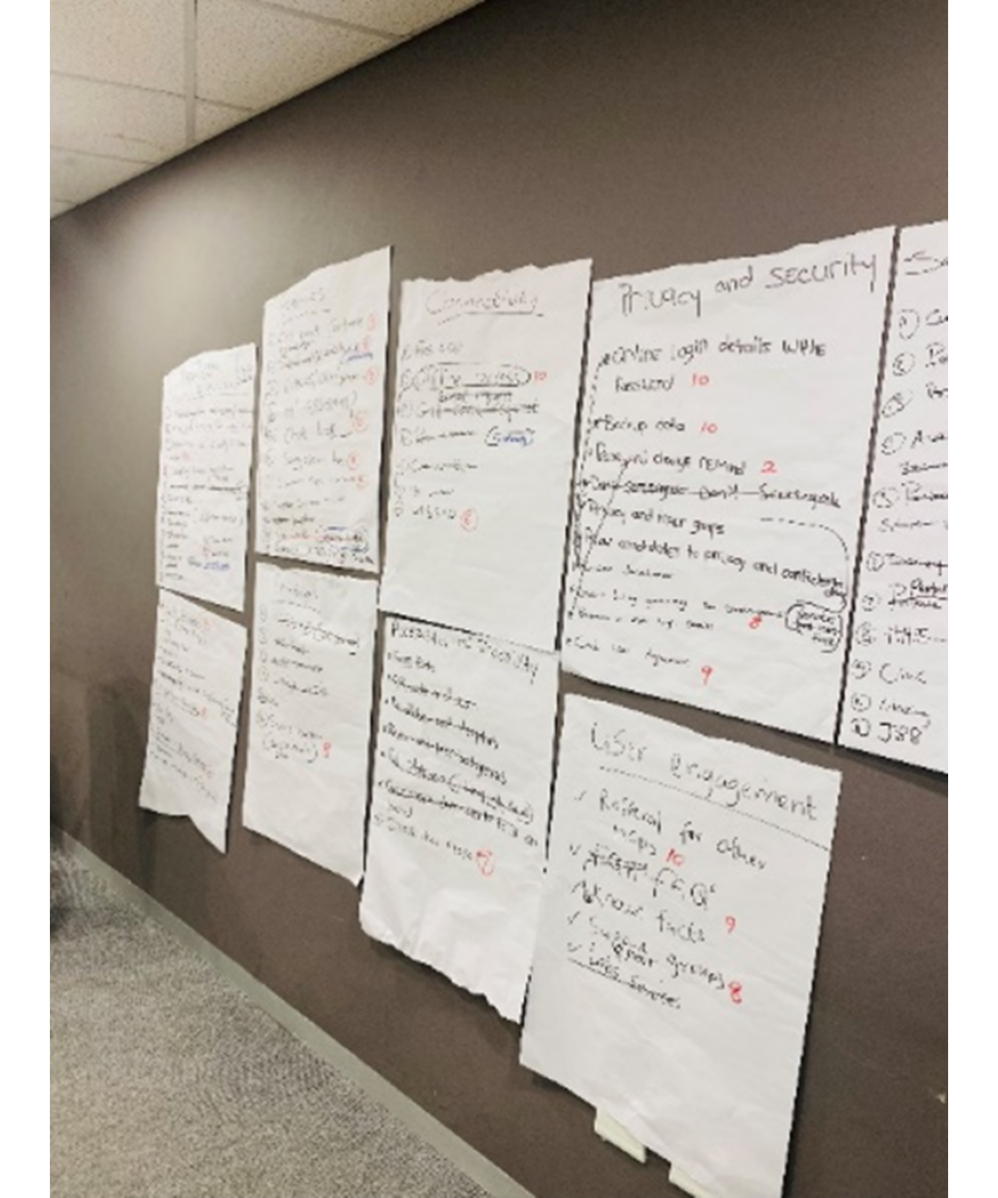
The process of ranking and voting on flipcharts.

The 2 research assistants collected the completed consent forms as well as all the ideas the NGT participants had written down on the sticky notes. The sticky notes were posted on the flipchart board. The principal investigator oriented the 2 research assistants on how the data needed to be classified around the guiding pillars. The principal investigator continued checking the work done by the research assistants to ensure that no idea was omitted in the flipchart recording (the generated ideas are shown in Figure 1). The second step after placing sticky notes on the flipchart was writing all the ideas using a black marker on different flipcharts with subheadings as themes (Figure 2). This enabled the experts to examine each idea generated and explain what it represented and vote and rank them accordingly (voting and ranking are shown in Figure 2). Finally, the principal investigator took down all the ranked and voted pillars, ideas, and subheadings, including the scores each had received (the findings are presented in Table 2).

Figure 2 depicts the ranking and voting on the flipcharts. These flipcharts were used during the ranking and voting by experts during steps 4 and 5 of NGT. Experts grouped similar ideas, ranked them according to priority, and voted for all contents that need to be included in the mHealth app per priority.

Figure 1 shows the initial generated ideas on sticky notes. These are the sticky notes collected from the experts after steps 1, 2, and 3 of NGT.

### Ethical Considerations

This study received ethics approval and clearance from the Sefako Makgatho Health Sciences Research Ethics Committee (SMUREC/H/291/2023:PG). All experts signed informed consent forms before the workshop commenced and were informed about their rights to withdraw from the workshop anytime they wished to without any consequences. The participants were allocated pseudonyms and numbers instead of their given names: E represented an expert, and 1 represented their allocated number; thus, all 13 experts had different numbers. All experts were reimbursed for transportation and fuel remuneration with 1000 ZAR (US $67; conversion rate 1 ZAR=US $0.067).

## Results

### Overview

In total, 13 experts participated in the NGT workshop meeting, and each expert represented different experiences, skills, and expertise. The 6 experts’ focus areas were in key populations and LGBTQIA+: 1 in innovative marketing, 1 in primary health care, 1 in data analytics, 1 in psychosocial science, 1 in SRH, 1 in communication, and 1 in obstetrics and gynecology and researcher (Table 1). Accordingly, the expert NGT workshop had a theoretical maximum ranking score of 13 for the pillars ranked, which comprised a total of 9 pillars with headings and subheadings. The experts focused on incorporating the pillars with similar and related functions. This was followed by identifying which headings resided under each pillar and then what information should be included as subheadings. Altogether, the experts voted and ranked the following pillars: menu options (66 points), privacy and security (39 points), user engagement (27 points), information hub (26 points), user demographics (20 points), connectivity (16 points), service integration and partnerships (10 points), functionalities (10 points), and accessibility and inclusivity (7 points; Table 2).

The content obtained through the NGT workshop is represented in Multimedia Appendix 1 by a tag cloud of themes to provide a high-level overview of the mHealth app, whereafter the specific themes and findings are discussed in detail. A tag cloud is a visual representation of text data in which the size of each word indicates its frequency or importance. Frequently used words appear larger and bolder, while less common words are smaller or lighter colored. Multimedia Appendix 1 presents the most prominent topics within the NGT workshop text. Data, information, services, support, privacy, and app are the most observably prominent themes.

### Menu Options

The menu options pillar was voted and ranked highest for inclusion in the mHealth app, with an overall score of 66 points. The experts emphasized the importance of this feature containing contact support information for queer individuals, with a ranking score of 11, followed by the mHealth app having a web-based consultation feature, with a ranking score of 11. The experts further suggested that mHealth should have an artificial intelligence (AI) assistant or chatbot, which ranked 10th, and a suggestion box, call-back, and email request features as important menu options, which ranked ninth each. Finally, the experts expressed that an mHealth app should accommodate videos and allow queer individuals to share videos by having a share button, both of which were ranked eighth. They further clarified that videos should contain interesting clips regarding queer matters and allow queer individuals to share success stories, such as successful gender transitioning and familial acceptance of queer children.

### Privacy and Security

The privacy and security pillar was the second top-voted and ranked pillar as experts emphasized the importance of the safety and security of queer individuals and their personal information when they used the mHealth app. Overall, this pillar received a score of 39 points, with an emphasis on ensuring the protection of the users’ data and safe record keeping, both with a ranking score of 10. In addition, the experts mentioned that these 2 headings should require passwords at log-in, and the mHealth app should have a backup database logging all the activities occurring on the mHealth app. The experts further voted for an mHealth app to contain an agreement section to ensure security, and this should provide an agreement between the users and the mHealth app developers before use, which scored 9 points. This was followed by ensuring users’ safeguarding with 8 points, where the experts highlighted that mHealth app developers should ensure the protection of users’ log-in details. The experts stressed the importance of the mHealth app having an option for allowing queer individuals to change their passwords regularly, with an embedded option of reminding them to change their passwords, achieving a ranking score of 2 points.

### User Engagement

The user engagement pillar followed the privacy and security pillar with a total of 27 points. This pillar had 3 headings, which the experts emphasized should be included in an mHealth app: ensuring referrals to other health professionals ranked 10 points; the availability of frequently asked questions ranked 9 points; and the availability of support groups in the mHealth app, where queer individuals can engage with other members with similar experiences and challenges, ranked 8 points. Furthermore, the experts clarified the importance of creating options in the mHealth app for queer individuals to meet peer groups; making known facts available for queer individuals in an mHealth app; and selecting or being referred to other health professionals for second opinions, advanced medical services, and specific queer-related services.

### Information Hub

The experts agreed on, ranked, and voted on the information hub after a long engagement and disagreement in terms of what this pillar should be called and what content it should include. An agreement was reached to call it an information hub, under which all health matters and topics apropos queer individuals would be placed. The information hub had an overall sum of 26 points. The experts emphasized the importance of including the health education information section, which had a ranking score of 11 points; followed by the gender-affirming services section, with 9 points; and lifestyle education, with 6 points. Moreover, a focus of the experts was that the health education section should include information such as HIV, postexposure prophylaxis, pre-exposure prophylaxis (PrEP), STIs, contraceptives, information on abortions, and where queer individuals could access and learn how to use lubricants and condoms and have the option of an indication of when to consult a physician when experiencing complications. Regarding gender affirmation, the experts stated that the information to be included should be about hormone and gender reassignment services. Finally, the experts highlighted the importance of information on sex toy use, their cleaning or care for hygiene purposes, and advice on healthy dietary practices.

### User Demographics

This pillar ranked 20 points in total. The experts pointed out that this pillar should have the ability to record personal data for the profile creation of queer individuals, which should include aspects such as their pronouns (ie, him, her, they, their, or them), their sexual identity (eg, male, female, binary, or nonbinary), age, and their gender orientation (eg, gay, bisexual, and transgender). This pillar had a ranking score of 11 points. Again, the experts emphasized the importance of an mHealth app having the ability to assist queer individuals in finding locations and health care facilities in different areas, focusing on ensuring that the mHealth app includes information on specialized health care facilities and nongovernmental organizations focusing on queer matters, which scored 9 points.

### Connectivity

The experts stressed the importance of how queer individuals should sign up for the mHealth app to access health care services and address the SRHSN of queer individuals, stating that an mHealth app should be free, which scored 10 points. The focus was ensuring that the mHealth app could be accessed offline (when users might not have data). Experts stated that the principal investigator should seek sponsorship of the mHealth app so that queer individuals could access it freely and whenever they are offline. Furthermore, the experts stated that the mHealth app should accommodate all queer individuals who do not have smartphones by providing the use of unstructured supplementary service data, with a ranking score of 6 points.

### Service Integration and Partnerships

The experts stressed the importance of an mHealth app being integrated and partnered with different health care departments to address SRHSN for queer individuals as one of the essential elements for inclusion. This had an overall voting and ranking sum of 10 points. Experts further mentioned that the mHealth app should be linked with other health services, such as HCPs, laboratories, medical aids, and private and public health care facilities, which would help with appointments and referrals.

### Functionalities

The experts discussed, voted, and ranked push notifications as functionalities that should be included in an mHealth app, with a ranking score of 10 points. The experts emphasized that this functionality would assist queer individuals with appointment reminders through SMS text messaging or emails, thereby improving their SRHSN.

### Accessibility and Inclusivity

This pillar was the lowest voted and ranked, with a score of 7 points. The experts accentuated that the anticipated mHealth app should not be about queer individuals only but also their families and cisgender individuals to help minimize stigma and enhance the acceptance and normality of queer existence. In addition, the experts stated that an mHealth app should accommodate people living with disabilities.

## Discussion

### Principal Findings

This paper presents a comprehensive description of suggestions made by a group of experts regarding the content that should be included in the development of an mHealth app to address queer individuals’ SRHSN. The proposed suggestions were based on the NGT steps followed during the workshop to ensure queer individuals’ SRHSN are addressed. On the basis of the NGT experts’ workshop, the crucial contents that need to be included in an mHealth app are represented by 9 pillars.

Our study reports on the experts’ suggestion for the anticipated mHealth app to contain various menu options, such as a web-based consultation feature, to enhance remote care. This suggestion aligns with several studies that established the need for mHealth apps to show and facilitate web-based patient-HCP interaction, as seen in a study by Cao et al [41] demonstrating that mHealth apps improve the exchange of 2-way information between patients and HCPs. In addition, our study echoes the results of the study by Markossian et al [42] that mHealth apps have interactive menu options that enable patients to engage immediately with physicians or nurses when experiencing challenges.

In addition, the experts in our study suggested that mHealth should have an AI assistant or chatbot feature to enable the provision of help for queer individuals and HCPs. Several studies identified similar findings to our study, such as the importance of using AI chatbots [43], the ease of using chatbots in supporting caregivers of people living with dementia, and the patients themselves [44]. Furthermore, a study by Haque and Rubya [45] found that chatbots are personalized, have humanlike engagement, and are received positively by users; however, much interest is lost due to improper responses and assumptions about users’ personalities. Aggarwal et al [46] found that participants view AI chatbots as a nonjudgmental space for engaging with sensitive information. Rossouw and Smuts [47] described conventional AI chatbot capabilities, such as keyword recognition and using prewritten texts and discussions to imitate conversational interaction. The same study also showed that conversational AI might be used to create unscripted AI chatbots, allowing for rapid and effective engagements that provide more tailored value to consumers [47]. Moreover, our study finds an emphasis on an mHealth app being accommodative and interesting by containing visuals and videos. Other studies note the inclusion of data visualizations [48] and video games [49] in mHealth apps to improve patients’ HCP engagement.

Our study emphasizes that privacy and security should be highlighted and included in the anticipated mHealth app. The experts participating in our NGT workshop highlighted the importance of safeguarding queer individuals’ personal data. They emphasized that the mHealth app should include a user agreement, provide secure log-in details such as passwords, and allow users to change their passwords regularly. A study by Liu et al [50] supported the outcomes reported in our study by indicating that mHealth app developers in China should pay attention to improving privacy protection processes. In addition, Schroeder et al [51] stated that people are hesitant to use mHealth apps because they have concerns about the security and privacy of their medical information. Again, our experts’ suggestions in this regard are achieved when using mHealth apps, which was also stated in a study by Zhou et al [52] as a barrier to using an mHealth app. Furthermore, the participants indicated they would require various security menu options in mHealth apps, such as regular password updates, remote wiping, user consent, and access control [52]. Finally, regarding privacy and security, Rezaee et al [53] proposed a comprehensive criterion for advising app developers, researchers, and designers by stating that passwords should be stronger and changed regularly.

Our study shows that if mHealth does not have sufficient information, queer individuals will not use it. As a result, an information hub pillar with SRHSN information, such as HIV, postexposure prophylaxis, PrEP, STIs, contraceptives, and information on abortions, was suggested by experts in our study. A study by del Río-Lanza et al [54] supports our findings, indicating that insufficient information was a barrier that limited participants’ use of the mHealth app. Several studies have produced various innovative solutions and interventions that addressed SRHSN for queer individuals; for example, Songtaweesin et al [55] focused on youth-friendly services and a mobile phone app to promote adherence to PrEP among adolescent men who have sex with men and transgender women at risk of HIV. Garg et al [56] promoted an mHealth app for self-education on HIV prevention and service; Tanner et al [57] examined the lessons learned from implementing the *weCare* intervention. Thus, mHealth apps are valuable tools that can be used for educational purposes [58].

The findings from our NGT expert workshop demonstrated the importance of connectivity, whereby queer individuals should be able to access an mHealth app free of charge, whether web-based or offline. It is indeed important for mHealth apps to be free, as evidenced in the study by Amiri et al [59], who stated that a lack of data and internet connection is a barrier to using these apps fully. Furthermore, in a similar study by Keel et al [60], the participants explained that the problem was weak internet connectivity. They further stated that they needed more data and stronger internet connections and affirmed that a strong and stable internet connection is key to mHealth app use [60]. Moreover, Karthan et al [61] stipulated that offline use should be a requirement (although this depends on the type of mHealth app) and further stated that, in general, sensor implementation should not require an internet connection. In addition, Tshimula et al [62] stated that the use of unstructured supplementary service data for electronic health records could enhance HCPs in managing and accessing patient information and communicating with health care systems in regions with poor internet connectivity.

Our NGT expert workshop ascertained that the mHealth app should accommodate service integration, allow referrals between HCPs, and remind queer individuals of their appointments and treatments. Ogundaini et al [63] corroborated our findings by stating that mHealth apps are appropriate for enhancing HCPs to consult with patients at hospital clinics. The same study shows that with mHealth, physicians can request detailed patient data from colleagues before making clinical decisions [63]. Furthermore, Steyn et al [64] highlighted that the *Vula* app is a useful tool for referring patients between departments; thus, it has the potential to improve the immediate quality of care and ensure progressive arrangements of care. Chao et al [65] concluded that mHealth apps should be able to provide reminders and alerts to patients. In addition, participants in a study by Nguyen et al [66] suggested that an mHealth app should be improved by adding scheduled reminders on phones (instead of delivering them via email) and incorporating them into the device’s native calendar. Finally, participants in the study by Peng et al [67] found reminders useful for busy people who were likely to forget things or those who needed reminders to take a lot of medication on a specific day.

### Strengths and Limitations

The development of an mHealth app to address SRHSN for queer individuals could be beneficial to such individuals, their family members, and HCPs to ensure and achieve inclusivity. Queer individuals would have access to SRHSN instantaneously by using the mHealth app through the phones in their pockets. Furthermore, HCPs’ skills and knowledge would be enhanced and strengthened, thus improving queer individuals’ well-being. Finally, queer individuals’ family members would attain an understanding of their loved ones without judgment or criticism but rather with openness and love through the knowledge they would receive from an mHealth app. We intended for our NGT experts’ workshop to have >25 participants, but 12 participants had to withdraw from the workshop due to work and family commitments. Considering that we had invited participants with significant work responsibilities and roles, we anticipated that this would be an obstacle. However, the experts who attended the workshop represented a vast array of specialties and expertise, as anticipated.

### Further Development and Research

The next step in intervention design is to develop an mHealth prototype in collaboration with software developers and engineers. This would provide us with a prototype that can be tested and evaluated by queer individuals, HCPs, and the group of experts who attended the workshop. The feedback from the testing will facilitate improving the prototype and developing a fully functional mHealth app. Future research should focus on innovative solutions that would address health care deficiencies for populations considered vulnerable such as queer individuals. In addition, an NGT should be used continually, as this will improve the quality of the work and redirect the focus toward determining what content should be included in other mHealth apps to address health-related issues.

### Conclusions

Using an NGT to advise and enhance the development of mHealth apps is crucial to improving health care services. The data provided by different experts and the discussions, arguments, and agreements helped gather information and feedback about what researchers should focus on. Our NGT expert workshop could serve as proof and evidence for scholars, developers, and designers who intend to create and develop innovative health care solutions to address the burden of diseases. In addition, the usefulness of a multidisciplinary approach and perspective to enhance the development of the mHealth app has shown that there is a need for the continued use of this method. Finally, an in-depth and proficient mHealth app is an approach to enhancing queer individuals’ SRHSN and well-being and ensuring equality and inclusivity.

## Appendix

Multimedia Appendix 1 Tag cloud of the nominal group technique themes and contents.

## Abbreviations

AI: artificial intelligence
HCP: health care provider
LGBTI: lesbian, gay, bisexual, transgender, and intersex
LGBTQIA+: lesbian, gay, bisexual, transgender, questioning, intersex, asexual, and related identities
mHealth: mobile health
NGT: nominal group technique
PrEP: pre-exposure prophylaxis
SRH: sexual and reproductive health care
SRHSN: sexual-reproductive health care services and needs
STI: sexually transmitted illness
WHO: World Health Organization

## Data Availability

The data sets generated during and analyzed during this study are available from the corresponding author on reasonable request.
