# Supplementary figures and images for: Development of an mHealth App by Experts for Queer Individuals’ Sexual-Reproductive Health Care Services and Needs: Nominal Group Technique Study
Source: JMIR Form Res. 2024 Aug 21;8:e59963. doi: 10.2196/59963 (PMC11375381; doi:10.2196/59963)

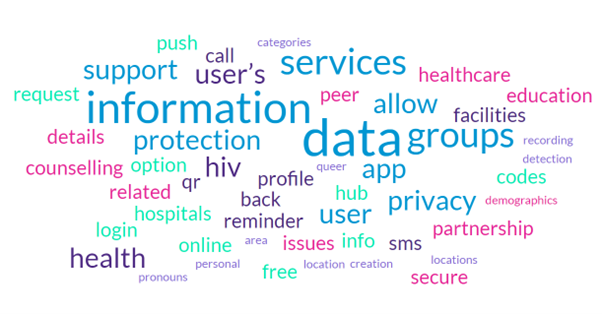

Supplement: Multimedia Appendix 1 [file formative_v8i1e59963_app1.png]
